# Native Heavy Metal-Tolerant Plant Growth Promoting Rhizobacteria Improves *Sulla spinosissima* (L.) Growth in Post-Mining Contaminated Soils

**DOI:** 10.3390/microorganisms10050838

**Published:** 2022-04-19

**Authors:** Malika Oubohssaine, Laila Sbabou, Jamal Aurag

**Affiliations:** Microbiology and Molecular Biology Team, Center of Plant and Microbial Biotechnology, Biodiversity and Environment, Faculty of Sciences, Mohammed V University in Rabat, Avenue Ibn Battouta, BP 1014, Rabat 10000, Morocco; malika.oubohssaine1@gmail.com (M.O.); l.sbabou@um5r.ac.ma (L.S.)

**Keywords:** abandoned mining sites, Plant Growth-Promoting Rhizobacteria, heavy metal-tolerant bacteria, inoculation, *Sulla spinosissima* (L.), antioxidant enzymatic activities

## Abstract

The potential of rhizobacteria in assisting plants used in the phytostabilization or re-vegetation of soils contaminated by heavy metals is gaining interest all around the world. In this context, six rhizobacterial strains isolated from highly heavy metal-contaminated soils situated in abandoned mining sites around the Oujda region (Morocco) were tested with *Sulla spinosissima* (L.), a native leguminous plant expanding in this area. The strains used were multi-resistant to heavy metals and possessed multiple plant growth-promoting traits. Potential beneficial effects of the strains were also evaluated in planta by measuring various growth and physiological parameters of inoculated *Sulla* plants grown in sterilized sand. Inoculation with the *Rhodococcus qingshengii* strain LMR340 boosted plant biomass (39% to 83% increase compared to uninoculated plants), chlorophyll and carotenoid content (up to 29%), and antioxidant enzyme activities (15% to 80% increase). Based on these interesting findings, selected strains were inoculated into plants growing in a heavy metal, multi-polluted, and poor soil. Under these conditions, non-inoculated plants and those inoculated with the strain LMR250 were unable to grow, while the other five bacterial inoculants restored plant growth. The best performing strain, *Pseudarthrobacter oxydans* LMR291, could be considered as a good biofertilizer and/or biostimulant candidate to be used for promoting the growth of selected plants in re-vegetation and/or phytostabilization programs of degraded and contaminated soils.

## 1. Introduction

World population growth and industrial activities are threatening the environment with the accumulation of different pollutants in soils and sediments. Soil contamination with toxic heavy metals has become a serious problem worldwide and one of the major causes of environmental deterioration. The inexpensive rehabilitation of contaminated soils can be attained through the employment of bacteria and vegetation that stabilize and/or extract metals from the soils, namely bioremediation and phytoremediation. They are considered as environmentally friendly alternative cleanup methods for decontaminating heavy metals or organic pollutants from the environment, particularly in agricultural farmlands, waste sites, or polluted waters [1,2]. These approaches are also considered economical and effective [3], and interestingly, they interfere less with the soil structure and they are more accepted by the public than other approaches or conventional techniques [1,4].

Rhizobacteria are among the most promising bacteria used in bioremediation and assisted-phytoremediation techniques. Rhizospheric bacteria are the principal actors, in addition to the roots and their exudates, in the functioning of the biosphere. Moreover, these bacteria, and especially those presenting positive effects on plant growth (PGPR), are considered as important potential tools for sustainable agriculture and a good choice for the reclamation of heavy metal-contaminated soils [5,6].

It is well-established that PGPR are very important for plant growth and health [7]. Plant growth promotion and development can be achieved directly through different mechanisms such as nitrogen fixation, inorganic phosphate solubilization, plant growth regulator production such as auxins, gibberellins and cytokinins, and the suppression of stress ethylene through 1-aminocyclopropane-1-carboxylate (ACC) deaminase activity [8]. PGPR can also have indirect effects on plants by preventing the growth or activity of plant pathogens through competition for nutrients and space, the inhibition of pathogen-produced enzymes or toxins, antibiosis, and the production of enzymes and the induction of plant defense mechanisms [9,10].

Besides their direct and indirect positive effects on biomass production, plant-associated bacteria can also contribute to an increase in metal availability and uptake, and to a decrease in metal phytotoxicity [11]. In recent years, PGPR have proven powerful for improving the phytoremediation of petroleum and other contaminants [12,13]. PGPR-assisted phytoremediation technology can provide better plant growth and remediate contaminated soils compared with using plants alone [14,15]. Moreover, soils exposed to long-term multi-pollution by organic compounds and/or heavy metals have been shown to contain large adapted microbial communities that can thrive under these harsh conditions [16]. This suggests that this type of microorganism may be superior for bioremediation and recovery of such contaminated sites. Abandoned mining sites are among the environments most polluted by heavy metals because of the considerable amounts of mining waste (waste from concentrates and waste rock) abandoned following the suspension of mining activities. In the absence of adequate management or rehabilitation programs, these sites can have very harmful effects on the environment and populations. This is the case of the abandoned mining sites studied, the Touissit-Sidi Boubker mining district and the Oued El Heimer foundry, which are located on the Algerian–Moroccan border south of the city of Oujda. Despite the region’s harsh climate and unfavorable soil conditions, several perfectly adapted plant species have been observed at these sites. Natural plants in this region, such as *Cistus libanotis* (L.), *Artemisia herba-alba* (L.), *Hirshfeldia incana* (L.), *Noea mucronata* (L.), *Lavandula dentate* (L.), *Phragmites communis* (L.), *Dittrichia viscosa* (L.) and others, are likely to have developed resistance to heavy metals and might be utilized in phytomanagement programs [17].

Among adapted plant species growing spontaneously in these polluted areas, legumes are considered as pioneer species that play an important role in the re-vegetation of the soils, especially because they can make soils healthy and fertile through root nitrogen fixing nodules [18]. *Sulla spinosissima* (L.) is one of the Mediterranean multi-tolerant legumes that are known for their ability to accumulate heavy metals in the roots and their contribution to soil fertility through the supply of nitrogen and other nutriments [19,20,21]. In Morocco, this species is naturally expanding in the abandoned mining sites situated in the Oujda region. Regarding all its properties, *S. spinosissima* (L.) can be an interesting model plant for studying bacterial-assisted phytoremediation.

In the present study, we have focused on rhizobacteria living with adapted plants in these heavy metal-polluted mining soils. Our principal objective was to select the best strains to be used in bacterial-assisted phytoremediation. For this purpose, a collection of bacteria was previously obtained from rhizospheric and the surrounding bulk soils of three plants growing in the region, the grass *Stipa tenuissima* (L.), the forage legume *Sulla spinosissima* (L.), and the legume tree *Acacia cyanophylla* (L.). All isolated bacteria passed through in vitro pre-screening tests concerning heavy metal tolerance and PGP traits and were identified by 16S rDNA sequencing. Results concerning the characterization of the bacteria isolated from the rhizosphere of *Sulla spinosissima* (L.) were published earlier by Oubohssaine et al. (2022) [22]. Among the strains that had showed promising properties, four were included in the present study along with two more strains from the *Stipa tenuissima* rhizobacterial collection. Before performing in planta tests by means of microbial inoculation of *Sulla spinosissima*, the characterization of those bacterial strains still uncharacterized was completed in vitro. Finally, their effect on heavy metal stress alleviation in *Sulla* plants was evaluated by growing inoculated plants in a heavy metal-contaminated soil. Differences between inoculated and non-inoculated plants were evaluated by measuring growth, photosynthetic parameters, and antioxidant enzymes activities.

## 2. Materials and Methods

### 2.1. Bacterial Strains

In order to select phyto-beneficial bacteria to be used as inoculants for plants targeted in phytoremediation approaches, we had previously constituted a collection of 701 isolates from soil samples of rhizospheric and surrounding bulk soils of three plants (*Acacia cyanophylla* (L.), *Sulla spinosissima* (L.), *Stipa tenuissima* (L.)). The three species are among the most abundant in the three abandoned mining sites, Touissit, Sidi Boubker, and Oued El Heimer. Except for *Acacia cyanophylla* (L.), which is an introduced legume tree species, the two others are herbaceous plants naturally growing in these soils. In an earlier publication, Oubohssaine et al. (2022) [22] reported results concerning the soil physico-chemical characteristics of the three sites. They were found to be highly and diversely contaminated by different heavy metals: chromium (Cr), copper (Cu), lead (Pb), zinc (Zn), and arsenic (As). In the same paper, some properties of bacterial isolates associated with *Sulla spinosissima* (L.), including their taxonomic status, their in vitro PGP activities, and heavy metal tolerance, were reported. The four most interesting strains, LMR266 (*Stenotrophomonas tumulicola*), LMR283 (*Pseudomonas brassicacearum*), LMR291 (*Pseudarthrobacter oxydans*), and LMR340 (*Rhodococcus qingshengii*), resulting from that work were selected to be tested in the present study [22]. In addition, we have included two strains isolated from *Stipa tenuissima* (L.) rhizosphere, LMR249 (*Pseudarthrobacter phenanthrenivorans*) and LMR250 (*Pseudarthrobacter oxydans*), which demonstrated interesting properties in preliminary studies (data not shown).

Before testing selected strains as inoculants of *Sulla spinosissima* (L.) plants under heavy metal stress conditions, it was important to ascertain the characteristics of the *Stipa* strains LMR249 and LMR250 by using appropriate quantitative methods and to compare them to the four *Sulla* strains selected from the previous study. In particular, the minimal inhibitory concentration (MIC) of each heavy metal was determined for each strain and the level of each PGP activity was quantified. In addition, biological nitrogen fixation potential of the six strains was assessed in vitro, and their phyto-beneficial effect measured in planta under non-stressed conditions.

### 2.2. Testing Resistance to Heavy Metals in Bacterial Isolates from Stipa Tenuissima Rhizosphere

Each strain’s minimal inhibitory concentration (MIC) for zinc, lead, and arsenic was determined. Bacteria were grown in triplicate on Nutrient Broth (NB) plates supplemented with increasing amounts of Zn, Pb, and As (5–35 mM). MIC was defined as the lowest metal concentration at which bacterial growth was not observed [23].

### 2.3. Evaluation of PGP Traits in Bacterial Strains from Stipa Tenuissima Rhizosphere

Quantification of four important PGP activities was performed following the detailed methods described by Oubohssaine et al., 2022 [22]: siderophore production by the chrome azurol-S (CAS) analytical method [24,25,26], natural phosphate solubilization by growing the strains in Pikovskays’ liquid medium [27,28], and determining free soluble phosphate by the vanadate–molybdate method [29], auxin production following the procedure of Gordon and Weber (1951) [30] and Sheng et al. (2008) [31], and ACC deaminase activity by using a modified assay from Penrose and Glick (2003) [32] and Li and Ramakrishna (2011) [33].

In addition, biological nitrogen fixation was assessed by the qualitative test described by Döbereiner (1995) [34]. In brief, strains were grown on semi-solid nitrogen-free medium seven times consecutively. After 96 h of incubation at 28 °C in the dark, the formation of a white growth film near the surface of the tubes indicated a positive result [35]. This procedure was performed in triplicate.

### 2.4. In Planta Evaluation of the PGP Traits of the Selected Strains

The phytobeneficial impact of the in vitro selected strains on the growth of *Sulla spinosissima* (L.) was assessed by growing inoculated plants in sterilized inert rock sand (sand autoclaved at 121 °C for 1 h).

Bacterial strains (LMR249, LMR250, LMR266, LMR283, LMR291, and LMR340) were cultivated overnight in NB medium and incubated at 28 °C at 180 rpm for 24 h. Bacterial cells were harvested by centrifugation at 8000 rpm at 4 °C for 10 min. Cell pellets were washed twice, re-suspended and adjusted with sterile distilled water in order to have an OD_600_ of 0.1 (equivalent to 10^8^ cells mL^−1^).

Seeds of *Sulla spinosissima* (L.) were collected from mining sites in the Oujda region. They were surface scarified manually, then sterilized with 70% ethanol for 1 min and rinsed five times with sterilized distilled water. Seeds were germinated on 0.9% agar plates at 25 °C in darkness for 4 days. Young seedlings were grown in small pots containing 100 g of sterilized sand (1 plant/pot and 12 repetitions/treatment). Each plant was inoculated with 1 mL of the corresponding bacterial inoculum. Uninoculated control plants were also planned. The experimental design was a randomized complete block. Pots were placed in a controlled growth chamber (16 h light, 8 h dark) with an average temperature of 28 °C. Pots were watered with a nutritive mineral solution [36] that contained K_2_HPO_4_ as the P source except for plants inoculated by the P-solubilizing strain LMR291 for which a natural phosphate powder was used (obtained by the fine grinding of rock phosphate from Khouribga mine (Morocco)).

#### 2.4.1. Measurement of Plant Biomass and Chlorophyll/Carotenoid Content

*Sulla* plants were harvested after 100 days of growth. Shoots were cut at the soil surface and roots were carefully separated from the sand or the soil. Roots and shoots were washed thoroughly with distilled water, and then dried with blotting paper. The length and dry biomass of both plants’ parts were measured.

The chlorophyll and carotenoid content in fresh leaves were estimated following the method of Mackinney (1941) [37]. One g of freshly cut leaves was ground to a fine pulp using a mortar and pestle after pouring in 2 mL of 80% acetone. The mixture was centrifuged at 5000 rpm for 5 min. The supernatant was collected, and its absorbance was read by a spectrophotometer at 645 and 663 nm for chlorophyll and at 480 and 510 nm for carotenoid against the blank (80% acetone).

The chlorophyll and carotenoid content present in the extracts of the leaves were calculated according to the equation given by Arnon (1949) [38]:

Total chlorophyll (mg g^−1^ leaf fresh mass) = (20.2 (OD_645_) + 8.02 (OD_663_)) × V/1000 × W (1)

Carotenoid (mg g^−1^ leaf fresh mass) = 7.6 (OD_480_) − 1.49 (OD_510_) × V/d × 1000 × W (2)

where OD_645_, OD_663_, OD_480_, and OD_510_ = Optical densities at 480, 663, 480, and 510 nm, respectively, V = Volume of an extract, W = Mass of leaf tissues, d = Length of light path (d = 1.4 cm).

#### 2.4.2. Antioxidant Enzyme Assays

Three different antioxidant enzymes activities (catalase (CAT), ascorbate peroxidase (APX), and peroxidase (POD)) were measured. Fresh tissue (0.3 g) was ground under chilled conditions with extraction buffer containing phosphate buffer (50 mM, pH 7.8), EDTA (0.1 mM), and 1% (*w*/*v*) PVP. The resulting homogenate was centrifuged at 12,000× *g* for 10 min at 4 °C and the supernatant collected for enzyme activity measurements. CAT, APX, and POD activities were measured according to Aebi (1984) [39] and Nakano and Asada (1981) [40]; Chen and Asada (1989) [41]; Chance and Maehly (1955) [42], respectively.

CAT activity was determined by spectrophotometry by following the decline in H_2_O_2_ at 240 nm as a function of time in a reaction mixture containing the enzymatic extract, 50 mM phosphate buffer (pH = 7), and 15 mM H_2_O_2_. The determination of the activity of this enzyme is calculated from the extinction coefficient ε = 0.036 mM^−1^ cm^−1^ and expressed in mM of H_2_O_2_ broken down per minute and per µg of protein.

APX activity was measured by following the decrease in the absorbance at 290 nm as a function of time caused by the oxidation of ascorbate in the presence of H_2_O_2_. The reaction mixture contained the enzymatic extract, 50 mM phosphate buffer (pH 7), 0.5 mM ascorbate, and 0.2 mM H_2_O_2_. The activity of this enzyme was calculated using the extinction coefficient ε = 2.8 mM^−1^ cm ^−1^.

Absorbance variations at 470 nm and 25 °C were used to calculate POD activity. The reaction was carried out in a 3 mL solution. A 10 µL amount of enzymatic extract was added to 2.99 mL of sodium phosphate buffer (50 mM, pH 6.0) containing the substrates (18.2 mM guaiacol and 4.4 mM H_2_O_2_). POD activity was defined as the quantity of enzyme that caused a 0.001 per minute rise in absorbance at 470 nm.

### 2.5. Effect of Inoculation with Selected PGPR Strains on Sulla Growth under Heavy Metal Stress Conditions

The highly heavy metal-contaminated soil of the Oued El Heimer site was used in this experiment. This soil is a low fertile alkaline soil that shows low levels of carbon, nitrogen, and phosphorus, and a low cation exchange capacity (CEC). Moreover, the soil is extremely polluted by five heavy metals (As, Cu, Zn, Cd, and Pb) (Oubohssaine et al. 2022) [22].

Young seedlings were grown in pots of the contaminated soil following the same experimental conditions reported in the precedent experiment, except for watering, which was performed with distilled water instead of a mineral solution. Bacterial inoculant preparation and delivery, as well as measured parameters, were also similar to previous experiments.

### 2.6. Statistical Analysis

Statistical analysis was conducted by using the Analysis of Variance (ANOVA) statistical package for social sciences XL STAT, followed by the comparison of multiple treatment levels, using the Duncan significant difference at *p* ≤ 0.05. Principal component analysis (PCA) was executed by using R software.

## 3. Results

### 3.1. Comparative Heavy Metal Resistance of Bacterial Strains Used as Inocula

Both strains isolated from the rhizosphere of *Stipa tenuissima* (L.) (LMR249 and LMR 250) showed, in general, moderate tolerance to the heavy metals tested. Only the strain LMR249 (*Pseudarthrobacter phenanthrenivorans*) showed a high level of tolerance to Zn with 10 mM as the minimal inhibiting concentration (MIC) (Table 1). For comparison, the four strains selected among those isolated from *Sulla* rhizosphere (LMR266, LMR283, LMR291, and LMR340) were more tolerant, with relevant results recorded for the *Rhodococcus qingshengii* strain LMR340 that was the most multi-resistant one with MIC values as high as 25, 30, and 35 mM, respectively, of Pb, Zn, and As. It was followed by LMR266 (*Stenotrophomonas tumulicola*) with 10, 20, and 10 mM MIC values, respectively.

### 3.2. PGP Activities in Bacterial Strains Used as Inocula

The quantitative method used for the estimation of siderophore production expresses the results in percentage compared to the negative control. The best production was recorded by the *Stipa* strain LMR250 (*Pseudarthrobacter oxydans*) with a value equal to 97.4 % (Table 1), followed by the *Sulla* rhizobacteria *Stenotrophomonas tumulicola*(LMR266) and *Pseudomonas brassicacearum*(LMR283),which showed siderophore production scores of 86.77% and 75.63%, respectively. The quantification of soluble phosphate produced in PVK liquid medium showed that the two *Stipa* rhizobacteria strains were less efficient in solubilizing natural phosphate than the best *Sulla* rhizosphere strain LMR291 that recorded the highest value (67.63 mg L^−1^).

Contrasting with P-solubilization results, the two *Stipa* rhizobacteria strains tested were able to produce high amounts of auxin in vitro, 127 and 66.66 µg mL^−1^, respectively, by the strains LMR250 and LMR249.However, the best auxin producers were the *Pseudomonas brassicacearum* LMR283 (144.98 µg mL^−1^) and *Pseudarthrobacter* LMR291 (134.15 µg mL^−1^) strains from the *Sulla* rhizobacteria collection. At the opposite, for ACC deaminase activity, higher values were obtained with the *Stipa* strain LMR249 (128 nmol mg^−1^ h^−1^ of α-ketobutyrate), followed by the *Sulla* rhizobacteria LMR291 and LMR340 (Table 1).

In addition to the PGP activities quantified, a qualitative test was used to estimate the biological nitrogen fixation potential of the six selected strains. Only three strains possessed the ability to fix atmospheric nitrogen freely, mainly isolates belonging to the species *Pseudarthrobacter oxydans* and *Rhodococcus* *qingshengii*.

### 3.3. Beneficial Effect of Selected Tolerant PGPR Strains on Sulla spinossisima (L.) Growing in Sterilized Sand

#### 3.3.1. Plant Growth Parameter Measurements

**Figure 1:**
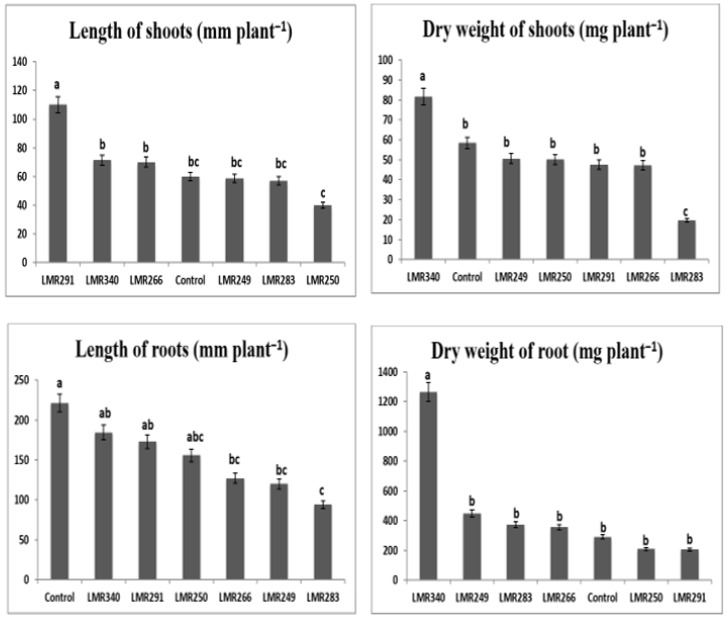
Effect of different bacterial inoculants on growth parameters of *Sulla* plants grown in sterilized sand. All the values are means of 12 replicates. One-way ANOVA was performed to determine the influence of inoculated bacteria on plant root and shoot biomass. Means for the different treatments with different letters are significantly different from each other (*p* < 0.05) according to the Duncan test.

The bacterial inoculation of *Sulla* plants growing in sterilized sand showed that the strain LMR340 (*Rhodococcus qingshengii*) had a beneficial effect on plant growth. Significantly higher shoot and root dry weights were recorded (81.74 and 1263.28 mg plant^−1^, respectively) compared with the the control plants (58.38 and 291 mg plant^−1^) and plants inoculated with the other bacterial strains (Figure 1). Another interesting strain, *Pseudarthrobacter oxydans* LMR291, induced the highest shoot length (110 mm plant^−1^) that was significantly different from all the other treatments including the control plants (60 mm plant^−1^). More interesting, is the fact that even if the plants inoculated with LMR291 were fertilized only with rock phosphate, their roots and shoot biomass were statistically equivalent to those of the control plants that received a soluble phosphate fertilizer.

It can be concluded that, in general, when compared with the control plants, most bacterial inoculants tested did not stimulate the growth of *Sulla* plants growing in sterilized sand, except for the *Rhodococcus qingshengii* strain LMR340 that had a net stimulating impact on plant biomass (Figure 1).

#### 3.3.2. Plant Chlorophyll and Carotenoid Content

**Figure 2:**
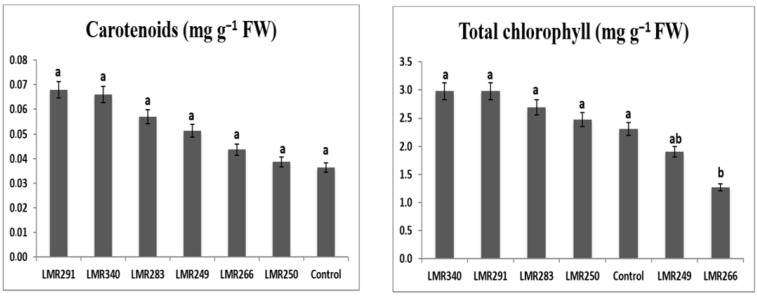
Total chlorophyll (mg g^−1^ FW) and carotenoid (mg g^−1^ FW) content of *Sulla* plants grown in sterilized sand conditions. Results are expressed as means ± SE (*n* = 3). One-way ANOVA was performed for each factor. Means with different letters are significantly different from each other (*p* < 0.05) according to the Duncan test.

*Sulla* inoculation with selected bacterial strains, especially LMR340 (*Rhodococcus qingshengii*) and LMR291 (*Pseudarthrobacter oxydans*), led to higher chlorophyll content (2.98 mg g^−1^ FW), with values statistically comparable to those of the control plants and those inoculated with the three other strains. Shoot carotenoid content also showed positive effects for all strains used as inoculants, but differences were not significant (Figure 2).

#### 3.3.3. Plant Antioxidant Enzymes Activities

Root POD activity was substantially greater in plants inoculated with LMR283, LMR266, and LMR250 (23.69, 17.32, and 16.10 U mg^−1^ proteins, respectively) than in the control plants (12.34 U mg^−1^ proteins). In leaves, POD activity was higher in plants inoculated with LMR340 and LMR249 (30.39 and 20.18 U mg^−1^ proteins, respectively) than in the control plants (14.75 U mg^−1^ proteins).

Root APX activity was markedly higher in plants inoculated with LMR283 and LMR266 (14.01 and 12.82 U mg^−1^ proteins, respectively) than in the control plants (7.83 U mg^−1^ proteins). In leaves, APX activity was substantially greater in plants inoculated with LMR340 or LMR283 (21.25 and 19.56 U mg^−1^ proteins, respectively) than in the control plants (12.53 U mg^−1^ proteins).

**Figure 3:**
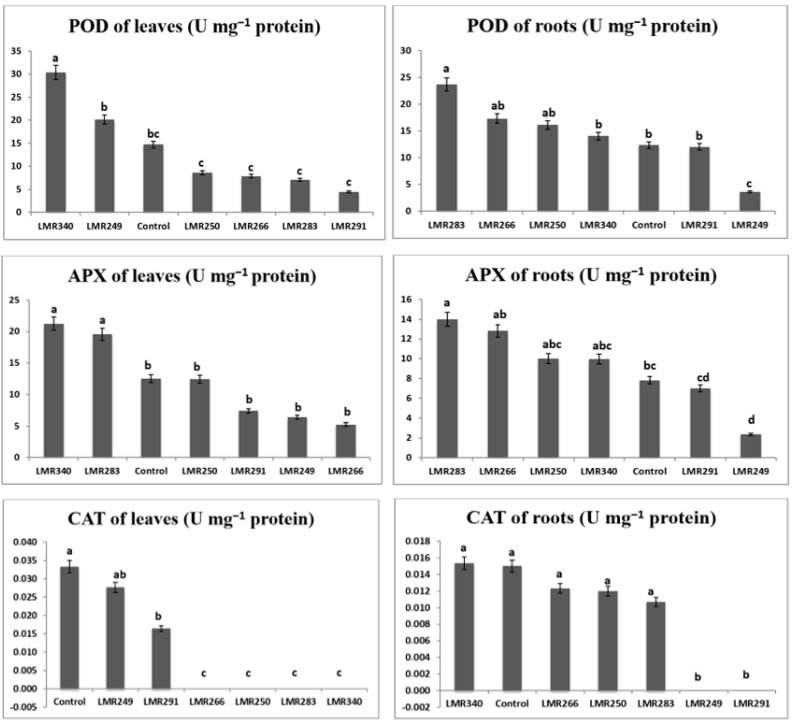
Changes in antioxidant enzymes in roots and leaves of *Sulla* plants 100 days after bacterial inoculation. All the values are means of 4 replicates. One-way ANOVA was performed to determine the influence of inoculated bacteria on antioxidant enzymes of plants growing in sterilized sand. Means for the different treatments with different letters are significantly different from each other (*p* < 0.05) according to the Duncan test.

The root CAT activities of plants inoculated with strains LMR340, LMR266, LMR250, and LMR283 were statistically equivalent to that of the control plants. It was relevant that plants inoculated with LMR291 and LMR249 didn’t show any root CAT activity. Plants inoculated with the last strains showed similar values to the control plants for CAT activity in leaves (0.02–0.03 U mg^−1^ proteins), whereas plants that were inoculated with strains LMR266, LMR250, LMR283, and LMR340 didn’t show any CAT activity (Figure 3).

#### 3.3.4. PCA Analysis

**Figure 4:**
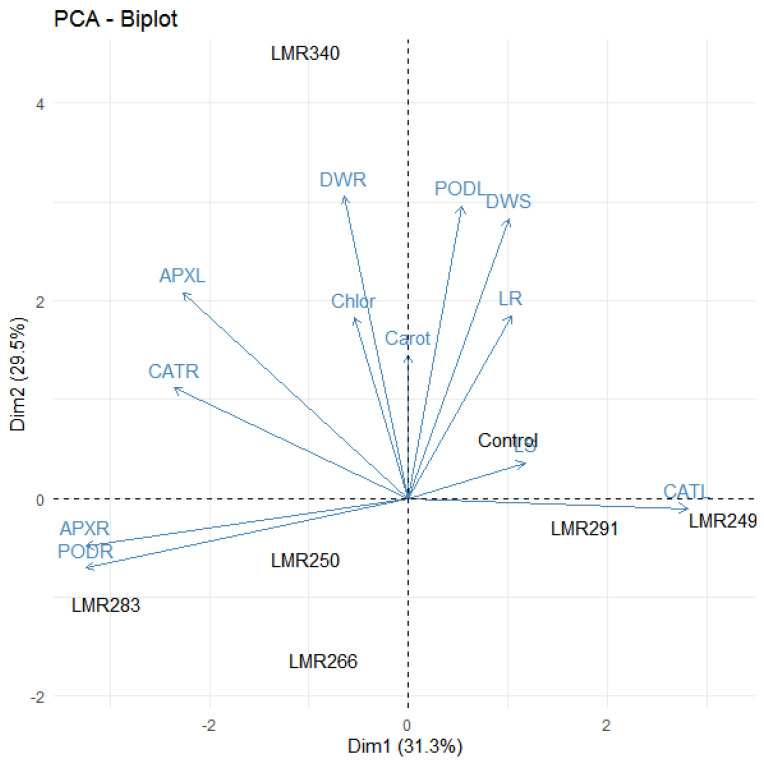
The correlation biplot between the Dim1 and Dim2 showed the 60.8% variation in which Dim 2 contributed 29.5% and Dim 1 contributed 31.3%. Strongly and positively correlated variables were very close to each other and present in the same quadrat. The blue color showed correlation between parameters (LS, LR, DWS, DWR, Chlor, Carot, PODL, PODR, APXL, APXR, CATL, CATR) while the black color showed correlation within treatments (Control, LMR249, LMR250, LMR266, LMR283, LMR291, LMR340).LS: Length of shoots, LR: Length of roots, DWS: Dry weight of shoots, DWR: Dry weight of roots, Chlor: Total chlorophyll, Carot: Carotenoids, PODL: POD of leaves, PODR: POD of roots, APXL: APX of leaves, APXR: APX of roots, CATL: CAT of leaves, CATR: CAT of roots.

Principal component analysis highlighted the positive effect of LMR340 on plant growth, chlorophyll and carotenoid content, and antioxidant enzyme activities. The variables which were strongly positively correlated were present in the same quadrant and very close to each other. For Dim 1, we noticed that PODL, DWS, LR, LS, and CATL were positively correlated, while DWR, Chlor, APXL, CATR, APXR, and PODR were negatively correlated. For Dim 2, all variables were strongly correlated except APXR, PODR, and CATL (Figure 4).

The PCA analysis showed that LMR340 was a singular inoculum compared to the other inoculation treatments.

### 3.4. Effect of Selected Heavy Metal-Tolerant PGPR Strains on Sulla spinosissima (L.) Growing in a Contaminated Soil

#### 3.4.1. Plant Growth Parameter Measurements

**Figure 5:**
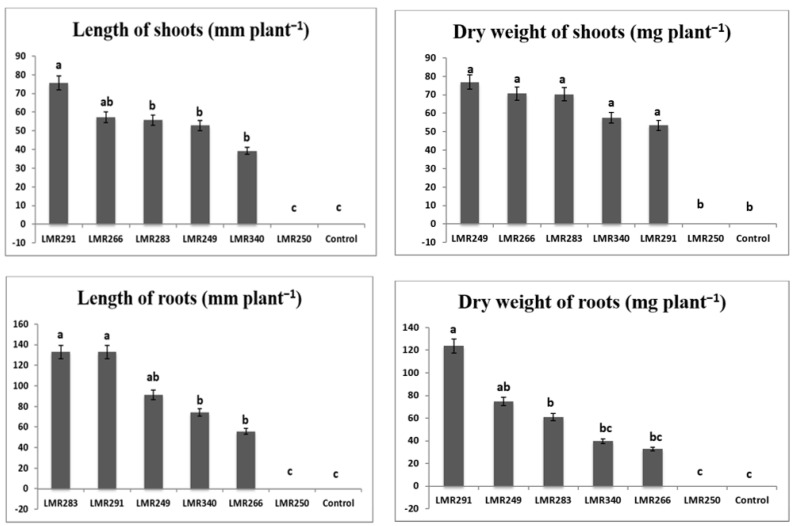
Effect of bacterial inoculation on shoots and roots of *Sulla* plants growing in heavy metal-contaminated soil. All the values are means of 12 replicates. One-way ANOVA was performed to determine the influence of inoculated bacteria on root and shoot biomass. Means for the different treatments with different letters are significantly different from each other (*p* < 0.05) according to Duncan test.

Except for the strain LMR250, inoculation with the heavy metal-tolerant PGPR bacteria (LMR291, LMR283, LMR249, LMR266, and LMR340) had a spectacular impact on plant growth in the highly multi-polluted soil used in this experiment, while non-inoculated control plants (T) failed to grow in such soil conditions and died quickly (Figure 5).

The results revealed that the strain LMR291 had the best impact on the growth of *Sulla* plants in the contaminated soil, with a significantly higher shoot and root lengths (75.71 and 132.85 mm, respectively) when compared to the other strains. Furthermore, root weight was significantly higher to that obtained with the other inoculant strains (123.84 mg plant^−1^), while all shoot weights were significantly equivalent except for the control plants and those inoculated with the strain LMR250.

#### 3.4.2. Plant Chlorophyll and Carotenoid Content

**Figure 6:**
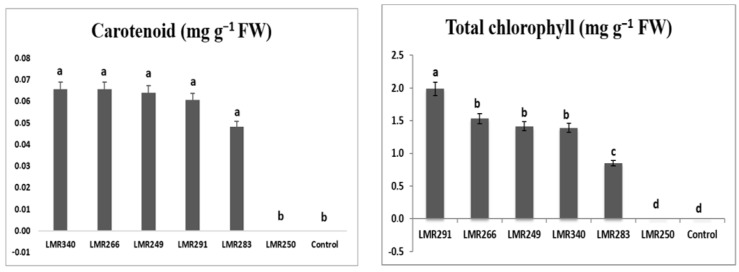
Total chlorophyll (mg g^−1^ FW) and carotenoids (mg g^−1^ FW) contents of *Sulla* plants growing in contaminated soils. Results are expressed as means ± SE (*n* = 3). One-way ANOVA was performed for each factor. Means with different letters are significantly different from each other (*p* < 0.05) according to the Duncan test.

Inoculation with LMR291 (*Pseudarthrobacter oxydans*) significantly enhanced the level of chlorophyll in *Sulla* plants growing under heavy metal stress compared with the other strains, while the plant carotenoid content was similar whatever the strain used as inoculum (Figure 6).

#### 3.4.3. Plant Antioxidant Enzymes Activities

Under metallic stress conditions, root POD activity results were significantly similar for all strains, except LMR340, which presented the lowest value of 7.51 U mg^−1^ proteins. For leaf POD activity, values were significantly higher in plants inoculated with LMR266 (7.57 U mg^−1^ proteins) and LMR340 (7.01 U mg^−1^ proteins) than in plants inoculated with LMR283, LMR249, and LMR291 (2.76, 2.32, 1.78 U mg^−1^ proteins, respectively), while non-inoculated control plants (T) and plants inoculated with LMR250 failed to thrive and died quickly in the multi-contaminated soil used.

The best results for root APX activity were obtained in plants inoculated with LMR266 (7.60 U mg^−1^ proteins) and LMR291 (6.52 U mg^−1^ proteins), whereas APX activity in leaves was much greater in plants inoculated with LMR249 than the other treatments.

**Figure 7:**
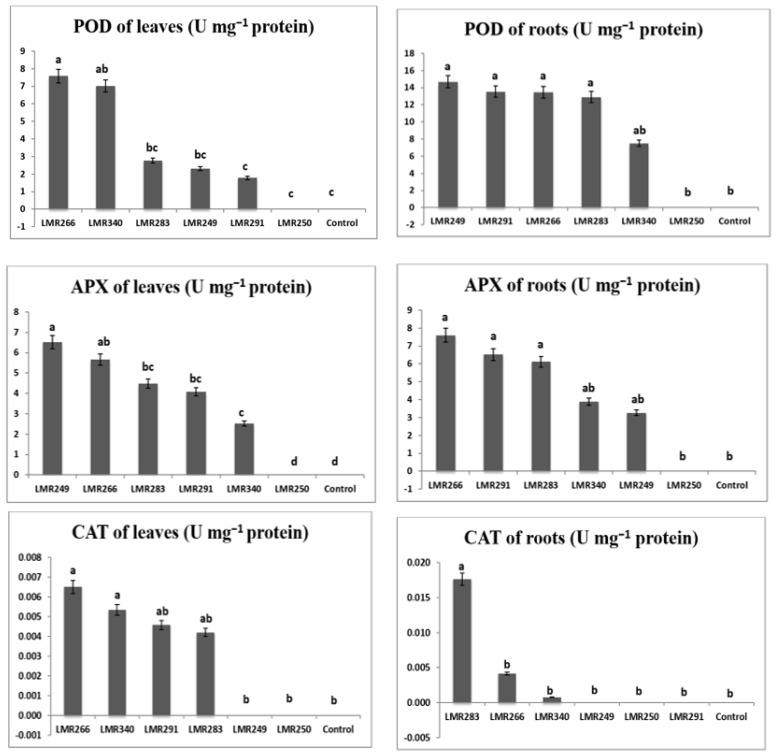
Antioxidant enzymes activities in roots and leaves of *Sulla* plants 100 days after bacterial inoculation. All the values are means of 4 replicates. One-way ANOVA was performed to determine the influence of inoculated bacteria on antioxidant enzymes for contaminated soil. Means for the different treatments with different letters are significantly different from each other (*p* < 0.05) according to the Duncan test.

In leaves, CAT activity was significantly higher in plants inoculated with LMR266, LMR340, and LMR291 (0.01 U mg^−1^ proteins) than plants inoculated with LMR249. In roots, CAT activity was significantly higher in plants inoculated with LMR283 (0.02 U mg^−1^ proteins) (Figure 7).

#### 3.4.4. PCA Analysis

**Figure 8:**
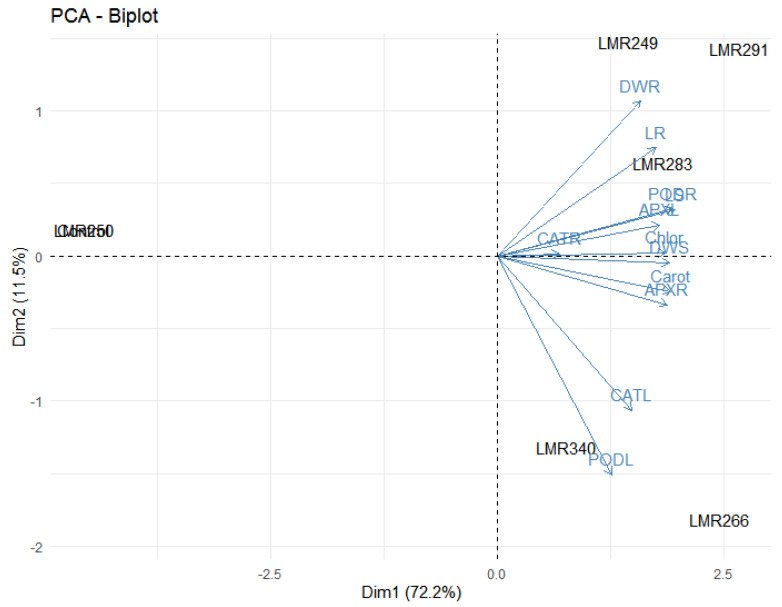
The correlation biplot between the Dim1 and Dim2 showed the 83.7% variation in which Dim 2 contributed 11.5% and Dim 1 contributed 72.2%. Strongly and positively correlated variables were very close to each other and present in the same quadrat. The blue color showed correlation between parameters (LS, LR, DWS, DWR, Chlor, Carot, PODL, PODR, APXL, APXR, CATL, CATR) while the black color showed correlation within treatments (Control, LMR249, LMR250, LMR266, LMR283, LMR291, LMR340). LS: Length of shoots, LR: Length of roots, DWS: Dry weight of shoots, DWR: Dry weight of roots, Chlor: Total chlorophyll, Carot: Carotenoids, PODL: POD of leaves, PODR: POD of roots, APXL: APX of leaves, APXR: APX of roots, CATL: CAT of leaves, CATR: CAT of roots.

It was relevant that all variables were strongly correlated for Dim 1, while for the second axis all variables were strongly correlated except PODL, CATL, APXR, and Carot. The PCA analysis indicated that LMR266 and LMR340 were special treatments in comparison with the others. On the other hand, LMR249, LMR291, and LMR283 were gathered at the same group, which can be explained by the fact that they possess the same characteristics (Figure 8).

## 4. Discussion

Plant PGPR-association benefits have been shown to include plant health and development, by suppressing microbial diseases and accelerating the accessibility and assimilation of nutrients [10]. Direct positive PGPR effects include plant growth regulator production such as auxin, phosphate solubilization, and the production of ACC deaminase [43]. Under stressful conditions, it would make more sense to use PGPR strains that are tolerant to prevailing stresses. In this context, the selection of heavy metal-tolerant PGPR strains is of paramount importance to improve plant growth and their resistance to heavy metal-contaminated soils for the implementation of effective strategies using bacterial-assisted phytoremediation.

In this study, we have compared the phytobeneficial potential of four *Sulla spinosissima* (L.) rhizobacterial strains (LMR266, LMR283, LMR291, and LMR340) characterized in vitro in a previous study [22], and two new strains isolated from *Stipa tenuissima* (L.) rhizosphere. The strains belong to four genera, *Pseudarthrobacter*, *Pseudomonas*, *Rhodococcus*, and *Stenotrophomonas*, and those isolated from *Sulla* rhizosphere are generally multi-resistant to high levels of the three heavy metals, Pb, Zn, and As [22]. The level of metal resistance is an important factor to be considered for heavy metal remediation since it is directly related to the survival of bacteria in metal-contaminated soils. In comparison to other reported metal-resistant strains, our bacterial isolates exhibited relatively higher metal resistance [44,45]. Additional to their heavy metal resistance, selected strains presented many PGP traits such as siderophore production. These iron-chelating molecules released by some rhizospheric bacteria to attract iron towards the rhizosphere [46,47], can promote plant health at different levels, especially by improving iron nutrition and preventing the growth of pathogens by limiting iron availability [48,49,50,51]. Additionally, they reduce the effects of metal pollution and help in phytoremediation processes by their capacity to bind other trace element ions [52]. In the current work, siderophores were produced by three strains: *Pseudarthrobacter oxydans* LMR250, *Stenotrophomonas tumulicola* LMR266, and *Pseudomonas brassicacearum* LMR283.

Among the heavy metal strains chosen for inoculation, the strains LMR250 and LMR291 (*Pseudarthrobacter oxydans*) and LMR340 (*Rhodococcus qingshengii*) possess the ability to fix molecular nitrogen. Nitrogen-fixing bacteria (NFB) provide natural nitrogen for the growth of native crops and plants [53,54] and can contribute significantly to the N budgets of a number of ecosystems [55]. In addition to nitrogen fixation, the strain LMR291 (*Pseudarthrobacter oxydans*), was a higher solubilizer of rock phosphate. Strains belonging to the *Pseudarthobacter* species were reported as phosphate solubilizers by many authors [56,57].

Auxin production is among the most sought after phyto-beneficial activity in PGPR strains to be used in stressful conditions [58,59,60]. In the present work, *Pseudomonas* (LMR283) and *Pseudarthrobacter* (LMR291, LMR250, LMR249), isolated from *Stipa* and *Sulla* rhizospheric soils, produced high amounts of auxin, which can be absorbed by inoculated plants, leading to an increase in their growth [61]. Moreover, 2 *Pseudarthrobacter* strains (LMR249 and LMR291) produced ACC deaminase, an enzyme that can promote plant growth under stressful conditions by lowering the ethylene levels in stressed plants [62,63].

On the whole, the six bacteria tested here were highly resistant to three heavy metals (Pb, Zn, and As) and possessed many important PGP traits, especially the strains LMR249, LMR291, and LMR340. Consequently, and as demonstrated by many authors for equivalent strains [64,65,66,67], it can be postulated that our strains are potential beneficial bacterial inocula that can enhance plant growth, positively influence the installation of a vegetative cover in heavy metal-contaminated soils and help to decrease or remove the metallic pollution.

To verify these hypotheses, in planta tests were performed using the species *Sulla spinosissima* (L.), which was chosen as a host plant regarding its capacity to grow spontaneously in highly and multi-heavy metal-contaminated soils in the Oujda mining district [19,22]. *Sulla* plants growing in sterilized sand were inoculated with the selected PGPR strains to verify if they have beneficial effects on plants. Results obtained showed that *Rhodococcus qingshengii* (LMR340) and, to a lesser extent, *Pseudarthrobacter oxydans* (LMR291), had positive impacts on plant growth, as well as chlorophyll and carotenoid content. These results can be correlated with the in vitro PGP properties of the strains, in particular, increased nutrient availability (N and/or P) through biological nitrogen fixation and inorganic phosphate solubilization, auxin production, and ACC deaminase activity. It was relevant that the plants inoculated with LMR291 and fertilized only with rock phosphate, had a biomass statistically equivalent to the control plants that received a soluble phosphate fertilizer. These results indicate that the solubilization activity of this strain measured in vitro was expressed in the rhizosphere of the plant and was sufficient to insure a normal growth level of *Sulla* plants. Inoculation with this strain could be a solution to enhance *Sulla* growth in mining soils, characterized by low total P content and reduced soluble P availability.

Levels of peroxidase (POD), ascorbate peroxidase (APX), and catalase (CAT) activities were variable depending on the strain inoculated, the plant organ, and the activity measured. The low increase in antioxidant enzymes activities measured in some cases could be attributed to stimulation by inoculated strains of plant defense mechanisms as suggested by Amna et al. (2020) [68].

The fascinating results obtained with *Sulla* plants inoculated with the selected strains prompted us to perform an experiment under stressful heavy metal circumstances to investigate the strains’ capacity to assist *Sulla* plants in surviving the high concentrations of metals found in contaminated soils. The mining soil of Oued El Heimer used in this experiment is characterized by toxic levels of heavy metals, especially As and Pb that were 2 and 10 times higher, respectively, than threshold values [22]. In this experiment, non-inoculated *Sulla* plants failed to grow under the extremely toxic soil conditions and died rapidly. In contrast, five heavy metal-tolerant PGPR strains, out of six tested, relieved soil heavy metal toxicity on *Sulla* plants (LMR291, LMR283, LMR249, LMR266, and LMR340).

The best positive impacts were attributed to the *Pseudarthrobacter oxydans* strain LMR291, which induced significantly higher lengths and weights of plant shoots and roots, and significantly enhanced the chlorophyll content of the plants over the other strains. However, compared with the non-stressed sterilized sand conditions, chlorophyll content of the plants was reduced by 30%. Heavy metals are known to reduce the contents of photosynthetic pigments, such as chlorophyll and carotenoids, mainly by affecting the cell wall and membrane integrity of thylakoids [69] and changes in proteins and DNA as a result of ROS interference [70,71,72]. Moreover, heavy metals such as Zn, Fe, Cu, Hg, Cr, and Pb can lead to an impediment of enzymes involved in the synthesis of chlorophyll, such as chlorophyll synthase and protochlorophyllide reductase [73]. However, several studies reported that inoculation with PGPR strains may enhance photosynthetic pigment content under heavy metal stress [74,75]. Mechanisms implicated include increasing nutrient uptake in plants through phosphate solubilization and the exudation of essential substances that play a crucial role in the synthesis of photosynthetic pigments [76]. The presence of carotenoids protects the plant’s photosynthetic machinery from photo-oxidative disruptions through ROS scavenging [77].

Many factors contribute to a strain’s potential to promote plant growth under heavy metal stress, including phosphate solubilization, IAA production, and nutrient availability [78,79]. In the current study, the strain LMR291 produced more auxin and ACC deaminase than the other strains tested, was able to solubilize phosphate, and tolerated three heavy metals (Pb, Zn, and As), which could explain why this strain, in particular, increased the biomass and photosynthetic activities of *Sulla* plants better compared to the other strains. The high capacity of LMR291 to solubilize inorganic phosphates in vitro is of great importance in the context of the post-mining soil used here that contained only 10 mg Kg^−1^ of available P [22]. The phosphate solubilization trait has a stimulant effect on photosynthetic activity, as noted by Demir (2005) [80] in his research. Phosphate also plays a crucial role in energy transfer in plants; hence, bacteria possessing this characteristic have a stimulating influence on photosynthetic activity. In addition, PGPR with various stress tolerance has the ability to improve plant tolerance to heavy metal stress by regulating hormone synthesis, antioxidant defense, and ethylene reduction, as well as aiding in stress-induced development [81].

The high performance of LMR291 could not be linked to the genus and species of this strain (*Pseudarthrobacter oxydans*) because another strain belonging to the same genus and species (LMR250) could not improve *Sulla* plant growth in the contaminated soil used. One possible explanation may be that the strain LMR291 was isolated from Oued El Heimer’s soil and the rhizosphere of *Sulla,* while the strain LMR250 was isolated from Sidi Boubker’s soil and *Stipa*’s rhizosphere. The adaptation of strains to soil conditions and plants seem to be important factors to be taken into consideration when selecting PGP strains for the inoculation of plants to be grown in contaminated soils. In the present study, the amount of Pb in soils seems to be a determining factor. Indeed, even if both soils were highly contaminated, the concentration of Pb in the soil of Oued El Heimer soil was the double of that measured in the Sidi Boubker soil [22]. Moreover, the strain LMR291 tolerated five times more Pb in vitro than the strain LMR250, and possessed superior levels of PGP activities, which are recognized as important traits for a strain to perform better with plants growing in heavy metal-contaminated soils (higher levels of P solubilization, auxin production, and ACC deaminase activity).

Plant exposition to metallic stress causes reactive oxygen species (ROS) production, hence, resulting in high oxidative damage [82]. Plants employ a detoxifying antioxidative system to maintain an optimum level of ROS, which includes different antioxidant enzymes, such as APX, CAT, and POD. These enzymes eliminate or balance the production of ROS in plants [83,84]. Antioxidant enzyme activities in plants under metal stress show variable trends depending on the metal concentration, duration of exposure, the metal ion, or plant species [85].

The comparison of the results obtained in our study under multi-heavy metal-contaminated conditions with other studies, such as [86,87], indicate that plants growing in the multi-polluted soil and inoculated with heavy metal-tolerant PGPR strains exhibited reduced antioxidant enzyme activities, especially in the aerial parts of the plants. However, inoculated plants maintained good levels of biomass, as well as chlorophyll and carotenoid content, compared to those of plants growing under the uncontaminated sterilized sand conditions. Thereby, the drop in antioxidant enzyme activities would not indicate that the plants are under stress, but rather would be attributed to the effect of tolerant PGPRs on the heavy metals in the rhizosphere, as reported by different studies [88,89,90,91,92].

Many previous reports highlighted the importance of the interaction between plants and tolerant PGPR in heavy metal-contaminated soils as they can increase the process of phytoremediation and plants can be protected from the injurious effects of metals [66,93,94,95]. The present work confirms that the inoculation with local selected heavy metal-tolerant PGPR strains enhanced the growth of *Sulla* plants in a very contaminated soil and, consequently, it can be considered as a valuable option to remediate contaminated soils.

However, even if the positive results of bacterial inoculation on plant growth are pronounced, there is a need to move on to more complex tests, especially under field conditions, where other factors such as climatic conditions may impact the performance of the bacteria, the plant, and their interaction. More globally, sustained efforts are still needed to make the inoculation of plants with bacteria an effective technique for bioremediation. In particular, it is essential to make this technique a highly reproducible and reliable process. This will require a deep understanding of the processes and interactions between bacteria, contaminants, soils, and plants.

## 5. Conclusions

The present work emphasizes the importance of selecting native heavy metal-tolerant strains possessing multiple plant growth-promoting traits during the process of finding bio-inoculants for selected adapted plants to be grown under conditions of metallic stress. Our results were particularly interesting given that the heavy metal-tolerant PGPR strains selected were able to restore *Sulla spinosissima* (L.) growth in a highly multi-polluted toxic soil. The strain LMR291 (*Pseudarthrobacter oxydans*), in particular, and to a lesser extent, LMR340 (*Rhodococcus qingshengii*), LMR249 (*Pseudarthrobacter phenanthrenivorans*), and LMR283 (*Pseudomonas brassicacearum*) substantially improved all the growth parameters of *Sulla* plants, their photosynthetic pigments, and their antioxidative enzymatic activities. Our results also improve our understanding of the mechanisms by which inoculated bacteria can alleviate plant heavy metal toxicity and suggest that PGPR inoculation could be an innovative approach for the phytomanagement of metal-contaminated lands around the world.

## Figures and Tables

**Table 1:** Heavy metal tolerance and plant growth-promoting traits of bacterial isolates under in vitro conditions.

Strain	LMR249	LMR250	LMR266	LMR283	LMR291	LMR340
Genus/species	*Pseudarthrobacter phenanthrenivorans*	*Pseudarthrobacter oxydans*	*Stenotrophomonastumulicola*	*Pseudomonas brassicacearum*	*Pseudarthrobacter oxydans*	*Rhodococcus qingshengii*
Sites	Sidi Boubker	Sidi Boubker	Sidi Boubker	Oued El Heimer	Oued El Heimer	Touissit
Origin	Rhizosphere of *Stipa tenuissima*	Rhizosphere of *Stipa tenuissima*	Rhizosphere of *Sulla spinosissima*	Rhizosphere of *Sulla spinosissima*	Rhizosphere of *Sulla spinosissima*	Rhizosphere of *Sulla spinosissima*
MIC of Pb (mM) *	5	5	10 ^†^	5 ^†^	15 ^†^	25 ^†^
MIC of Zn (mM) *	10	5	20 ^†^	10 ^†^	3 ^†^	30 ^†^
MIC of As (mM) *	5	5	10 ^†^	5 ^†^	5 ^†^	35 ^†^
P (mg L^−1^) *	2.31 ± 0.1	0	0	2.44 ± 0.3 ^†^	67.63 ± 0.1 ^†^	0
Quantity (nmol mg^−1^ h^−1^) of α cetobutyrate (ACC Activity) *	128 ± 0.8	4 ± 0.5	0	0	71.1 ± 1.4 ^†^	58 ± 2.3 ^†^
Auxin production (µg mL^−1^) *	66.66 ± 1.6	127 ± 0.1	19.5 ± 1 ^†^	144.98 ± 0.9 ^†^	134.15 ± 0.6 ^†^	26.67 ± 1.2 ^†^
Siderophore production (%) *	59.71 ± 2.3	97.4 ± 1.4	86.77 ± 0.9 ^†^	75.63 ± 2.4 ^†^	0	0
Nitrogen fixation	-	+	-	-	+	+

MIC, minimum inhibitory concentrations; ACC, 1-aminocyclopropane-1-carboxylate (ACC) deaminase; Auxin, indole-3-acetic acid; P, Rock phosphate solubilization; +, presence of the trait; -, absence of the trait, *: Values are the average of three repetitions ± standard errors. **^†^**: Data from a previous work [22].

## Data Availability

Authors confirm that all relevant data are included in the article. Materials are available from the corresponding author upon reasonable request.
